# Exposing the deadly dark side of apoptotic cancer stem cells

**DOI:** 10.18632/oncoscience.369

**Published:** 2017-10-23

**Authors:** Goodwin G. Jinesh

**Affiliations:** Department of Urology, University of Texas MD Anderson Cancer Center, Houston, Texas 77030, USA

**Keywords:** metastasis, reactive oxygen species, K-Ras, Hemoxygenase-1/HO-1, PKC-ζ

**Figure 1:**
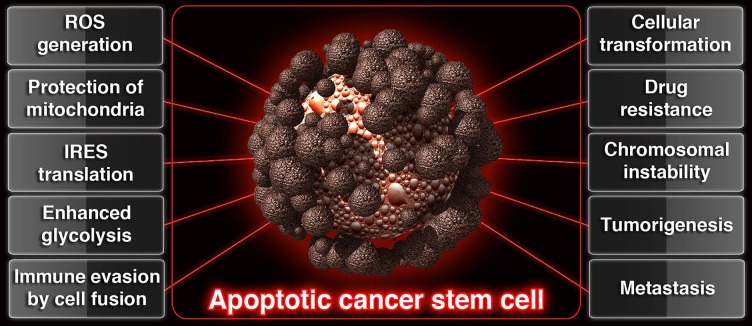
Schematic showing the contribution of apoptotic cancer stem cells to various hallmarks of cancer

Under physiological conditions *in vivo*, apoptotic cells are broken down and packed as smaller apoptotic bodies so that, phagocytes can engulf and digest apoptotic bodies by phagocytosis. However, recent studies point out the fact that cancer stem cells which undergo morphological and biochemical apoptosis [1], construct blebbishields from apoptotic bodies using serpentine filopodia in a dynamin- dependent endocytosis-dependent manner [2], override phagocytosis by blebbishield-immune cell fusion [3] to undergo cellular transformation by blebbishield-blebbishield or blebbishield-mitotic cell or blebbishield-immune cell fusion [1–3], and form tumors in mice [1, 3]. These exciting new features of blebbishield emergency program linked apoptotic cancer stem cells to drug resistance [1], immune evasion [3], apoptosis evasion [1, 4], tumorigenesis [1, 3], enhanced glycolysis [4] generation of chromosomal instability [3], increase in nuclear size [3], and metastasis [3] (Figure 1). Hence apoptosis is an adventure trip for cancer stem cells rather than a beginning of their own destruction and elimination by phagocytes.

How do the cancer stem cells master the art of surviving apoptosis? Although reactive oxygen species (ROS) can induce apoptosis, ROS has the answer for this question because, K-Ras, PKC-ζ and p47phox mediated ROS generation drives blebbishield emergency program [5, 6]. ROS keep the PKCs active and PKCs in turn activate p70S6K [6] to regulate internal ribosome entry site (IRES)-dependent translation of anti-apoptotic factors during the progression of apoptosis [4]. Inhibitor of apoptotic proteins such as c-IAP2, XIAP and critical molecules for transformation such as VEGF-A, and N-Myc are under the control of IRES translation [2, 4]. Thus the pro-apoptotic versus anti-apoptotic balance shifts towards survival. In addition to ROS generation, the apoptotic cancer stem cells also protect their mitochondria from depolarization using Pim-1 kinases [7, 8] to continue performing glycolysis, and producing ROS.

How do the cancer stem cells master the art of evading phagocytosis and initiating cell fusion? In fact, apoptotic cancer stem cells (blebbishields) evade phagocytosis by cell fusion with immune cells to interfere with clonal deletion of immune cell-blebbishield hybrid cells and result in hepatosplenomegaly [3]. Hence cell fusion drives phagocytosis evasion. Cell fusion is driven by serpentine filopodia generated by dynamin-dependent endocytosis [2]. Hence dynamin-dependent endocytosis precedes cell fusion and phagocytosis evasion. Endocytosis is initiated in apoptotic cancer stem cells by caspase-3-mediated cleavage of β-catenin to release cleaved 72-kDa β-catenin/K-Ras/PKC-ζ/cdc42/VEGFR2 from E-cadherin [2]. Thus initiation of endocytosis during apoptosis by caspase-3 is the key to trigger phagocytosis evasion cascade.

How endocytosis contributes to filopodia formation in apoptotic cancer stem cells to enable cell fusion? When caspase-3 initiates endocytosis, cdc42 a major filopodia nucleating/generating factor is also released from E-cadherin-mediated lock [2]. Furthermore, cdc42 [2], p70S6K [2, 4], hemoxygenase-1 (HO-1) [3], and VEGFR2 [1–3] are well-known to play major roles in blebbishield emergency program and are also known to localize at filopodia to regulate filopodia activity. Filopodia in-turn promotes membrane apposition and adherent junction formation to promote cell fusion by forming adhesion- zippers using filopodia from opposite membranes [2].

Thus the apoptotic cancer stem cells has lethal roles to play by promoting K-Ras activation, protection of mitochondria by Pim-1 kinase, glycolysis, ROS generation, PKC-ζ activation, p70S6K activation, IRES translation of anti-apoptotic factors, dynamin-dependent endocytosis, serpentine filopodia formation, cell fusion, cellular transformation, drug resistance, tumorigenesis, chromosomal instability, nuclear size increase, and metastasis.
